# AMPK Suppresses Connexin43 Expression in the Bladder and Ameliorates Voiding Dysfunction in Cyclophosphamide-induced Mouse Cystitis

**DOI:** 10.1038/srep19708

**Published:** 2016-01-25

**Authors:** Xiling Zhang, Jian Yao, Kun Gao, Yuan Chi, Takahiko Mitsui, Tatsuya Ihara, Norifumi Sawada, Manabu Kamiyama, Jianglin Fan, Masayuki Takeda

**Affiliations:** 1 Department of Molecular Signaling, Interdisciplinary Graduate School of Medicine and Engineering, University of Yamanashi, Yamanashi, Japan; 2 Department of Urology, Interdisciplinary Graduate School of Medicine and Engineering, University of Yamanashi, Yamanashi, Japan; 3 Department of Molecular Pathology, Interdisciplinary Graduate School of Medicine and Engineering, University of Yamanashi, Yamanashi, Japan; 4 Department of Urology, The 4th affiliated hospital of China Medical University, Shenyang, China

## Abstract

Bladder voiding dysfunction is closely related to local oxidation, inflammation, and enhanced channel activities. Given that the AMP-activated protein kinase (AMPK) has anti-oxidative, anti-inflammatory and channel-inhibiting properties, we examined whether and how AMPK affected bladder activity. AMPK activation in rat bladder smooth muscle cells (BSMCs) using three different AMPK agonists resulted in a decrease in connexin43 (Cx43) expression and function, which was associated with reduced CREB phosphorylation, Cx43 promoter activity and mRNA expression, but not Cx43 degradation. Downregulation of CREB with siRNA increased Cx43 expression. A functional analysis revealed that AMPK weakened BSMC contraction and bladder capacity. AMPK also counteracted the IL-1β- and TNFα-induced increase in Cx43 in BSMCs. *In vivo* administration of the AMPK agonist AICAR attenuated cyclophosphamide-initiated bladder oxidation, inflammation, Cx43 expression and voiding dysfunction. Further analysis comparing the responses of the wild-type (*Cx43*^+/+^) and heterozygous (*Cx43*^+/−^) Cx43 mice to cyclophosphamide revealed that the *Cx43*^+/−^ mice retained a relatively normal micturition pattern compared to the *Cx43*^+/+^ mice. Taken together, our results indicate that AMPK inhibits Cx43 in BSMCs and improves bladder activity under pathological conditions. We propose that strategies that target AMPK can be developed as novel therapeutic approaches for treating bladder dysfunction.

Voiding dysfunction refers to an abnormality in filling or emptying the bladder, which occurs in many pathological situations, including interstitial cystitis/bladder pain syndrome, overactive bladder (OAB), incontinence, obstruction of the urinary tract and urination difficulties due to neurological conditions or spinal cord injury. Among the various symptoms of voiding dysfunction, urinary frequency is the most common. It is not lethal, but it severely affects the quality life of the patients^1^,^2^,^3^. Currently, there are limited therapeutic options available to eradicate this symptom.

The pathological mechanism of frequent urination in humans remains elusive. Animal experiments have shown that it could be due to an enhanced bladder response to chemical and mechanical stimuli, such as neurotransmitters, growth factors, inflammatory mediators, hydraulic pressure and fluid shear stress. Multiple molecular mechanisms may be involved. Several lines of evidence, mainly from animal studies, implicate altered channel activities, including transient receptor potential channels (TRP channels), Ca^2+^ released channels, ATP receptor-operated channels and gap junction (GJ) channels^4^,^5^,^6^,^7^,^8^,^9^. Among these channels, gap junctions (GJs) have been extensively investigated and are recognized as one of the key factors influencing bladder activity. GJs, which are formed by a family of specific proteins termed connexins (Cxs), are intercellular channels that permit the exchange of ions, second messengers, and small signaling molecules between adjacent cells and play a pivotal role in the control of tissue hemostasis and cellular function^10^,^11^. Bladder smooth muscle cells (BSMCs) express several different isoforms of Cxs, including Cx26, Cx37, Cx40, Cx43 and Cx45^12^,^13^. The channels formed by these Cxs serve as pathways for intercellular transmission and the propagation of electrical signals, contributing to the coordinated contraction and relaxation responses required for bladder emptying and filling^14^,^15^,^16^. Most of the studies on GJs in the bladder focus on Cx43, a molecule that is ubiquitously expressed in almost all cell types^12^,^13^,^14^,^15^,^16^. Clinical and experimental studies demonstrated a close association between elevated Cx43 expression and detrusor overactivity^13^,^14^,^15^,^17^,^18^,^19^. The pathological factors implicated in urinary disorders, such as oxidative stress, inflammatory mediators and growth factors, have been documented to be able to upregulate Cx43 expression^15^,^20^,^21^,^22^,^23^,^24^,^25^. Because of these reasons, targeting Cx43 has been proposed as a potential approach to treat bladder hyperactivity. However, this idea has not yet been tested. The available approaches for inhibiting Cx43 expression in the bladder are still limited. It is highly desirable to identify strategies that can modulate Cx43 expression in the bladder and alter bladder activity.

AMPK is a serine/threonine kinase that functions as an intracellular energy sensor in eukaryotic cells to maintain cellular energy homeostasis. AMPK is activated under low cellular energy status. Once activated, it activates ATP-generating catabolic pathways and inhibits ATP-utilizing anabolic pathways. In addition to its well-reported regulatory effects on metabolism, AMPK also has anti-inflammatory, anti-oxidative, and channel-inhibiting properties^26^,^27^,^28^,^29^,^30^,^31^,^32^,^33^,^34^. Several findings prompted us to speculate that AMPK might have the potential to regulate Cx43 expression in the bladder and likely bladder activity as well. First, AMPK counteracts the biological actions of several inflammatory mediators and growth factors, such as interleukins, tumor necrosis factor, PDGF, etc., including those implicated in upregulation of Cx43 expression in the bladder and overactivity of bladder^21^,^22^,^28^,^30^. Second, metabolic syndrome has emerged as a major risk factor for voiding disorders, while AMPK activation is an effective therapeutic strategy against metabolic disorders^26^,^35^,^36^. Third, as a sensor of energy status, AMPK activation in response to metabolic stress shuts off several energy-consuming channels. Thus, GJs, an integrated part of nutrient transport system, should be affected by AMPK. Fourth, autophagy, which is known to be activated by AMPK, has been recently described as a pathway for Cx43 degradation^37^,^38^. Therefore, we designed experiments to explore the potential roles and mechanisms of AMPK in the regulation of Cx43 expression in the bladder and bladder function.

Here, we present our results that AMPK downregulates Cx43 expression and influences micturition under both physiological and pathological conditions. Thus, our study indicates that treatments targeting AMPK can be developed as new therapeutic strategies to alleviate bladder overactivity.

## Results

### AMPK suppresses Cx43 expression and function

**Figure 1:**
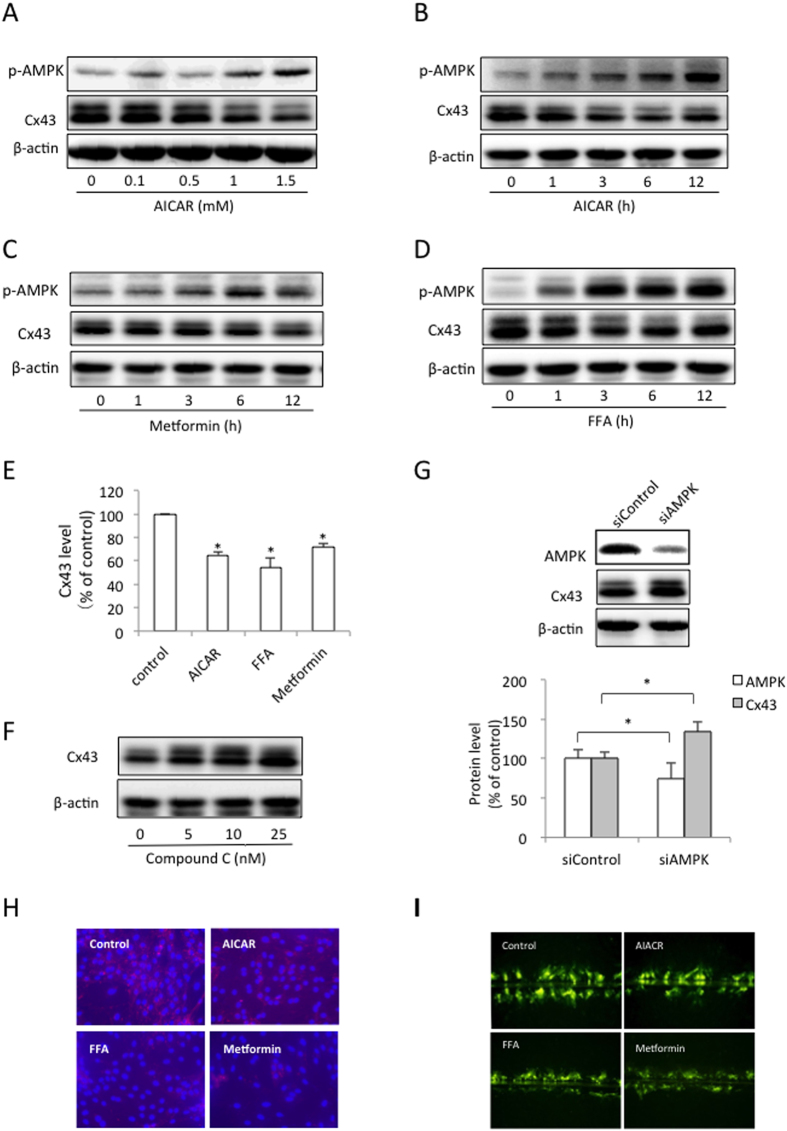
AMPK suppresses Cx43 expression and function. (**A**–**E**) AMPK activation suppresses Cx43 expression. The BSMCs were exposed to various concentrations of AICAR (**A**) for 12 h, or 1 mM AICAR (**B**), 5 mM metformin (**C**) and 50 μM FFA (**D**) for the indicated times. The cellular proteins were extracted and subjected to Western blot analysis for phosphorylated AMPKα (p-AMPKα) and Cx43. (**E**) Densitometric analysis of the Cx43 levels after the cells were treated with the AMPK agonists. The BSMCs were exposed to 1 mM AICAR, 50 μM FFA, or 5 mM metformin for 12 h. The intensity of the Cx43 signal was measured using ImageJ software. The data represent the mean ± SEM of 3 independent experiments comparing the untreated cells and those stimulated with AICAR, FFA or metformin (*p < 0.01 versus control, one-way ANOVA followed by Dunnett’s test). (**F**,**G**) AMPK inhibition increases Cx43 expression. The BSMCs were exposed to various concentration of Compound C for 12 h (**F**) or transfected with a specific siRNA against AMPK for 48 h (**G**). The cellular proteins were subjected to Western blot analysis for Cx43 and AMPK. A quantitative analysis of the effect of the AMPK siRNA on the levels of AMPK and Cx43 is shown in the lower panel of (Figure **G**). The results are expressed as the fold induction relative to the basal levels of AMPK and Cx43. The data represent the mean ± SEM of 3 independent experiments comparing the siControl and siAMPK cells (^*^p < 0.05, two-tailed t-test). (**H**) Effect of the AMPK agonists on the Cx43 distribution. The BSMCs were either untreated or incubated with 1 mM AICAR, 50 μM FFA, or 5 mM metformin for 12 h, and then subjected to Cx43 (red) and nuclear (blue) staining. (**I**) Effect of AMPK activation on gap junction intercellular communication. The BSMCs were treated the same as above. The micrographs of LY (green) diffusion into the cellular monolayer after scrape loading are shown (magnification, ×200).

We stimulated BSMCs with three different AMPK agonists to determine the potential role of AMPK in Cx43 expression. As shown in Fig. 1A,B, the BSMCs that were exposed to the well-used AMPK agonist 5-aminoimidazole-4-carboxamide-1-β-D-ribofuranoside (AICAR) exhibited a time- and concentration-dependent reduction in Cx43 expression. Two other AMPK agonists, metformin and FFA, inhibited Cx43 similarly (Fig. 1C,D). The quantitative analysis indicated that incubating the BSMCs with 1 mM AICAR, 50 μM FFA or 5 mM metformin for 12 h led to a significant suppression of Cx43 protein expression (Fig. 1E). Accordingly, suppression of AMPK with a chemical inhibitor, Compound C, or a specific siRNA significantly enhanced Cx43 protein expression (Fig. 1F,G). These results suggest that AMPK activation inhibits Cx43 expression.

Immunofluorescent staining of Cx43 with a specific antibody revealed that the punctate Cx43 spots were localized at the perinuclear and cell-to-cell contact regions. AMPK activation caused an obvious reduction in the C43 protein in both locations (Fig. 1H).

To determine whether Cx43 inhibition was associated with reduced gap junctional intercellular communication (GJIC), we performed the scrape loading dye transfer assay. AICAR, FFA and metformin all inhibited dye transfer from the scrape-loaded cells to the neighboring cells (Fig. 1I). Collectively, these results indicate that AMPK activation suppresses Cx43 expression and function.

### AMPK regulates BSMC contraction and bladder activity

**Figure 2:**
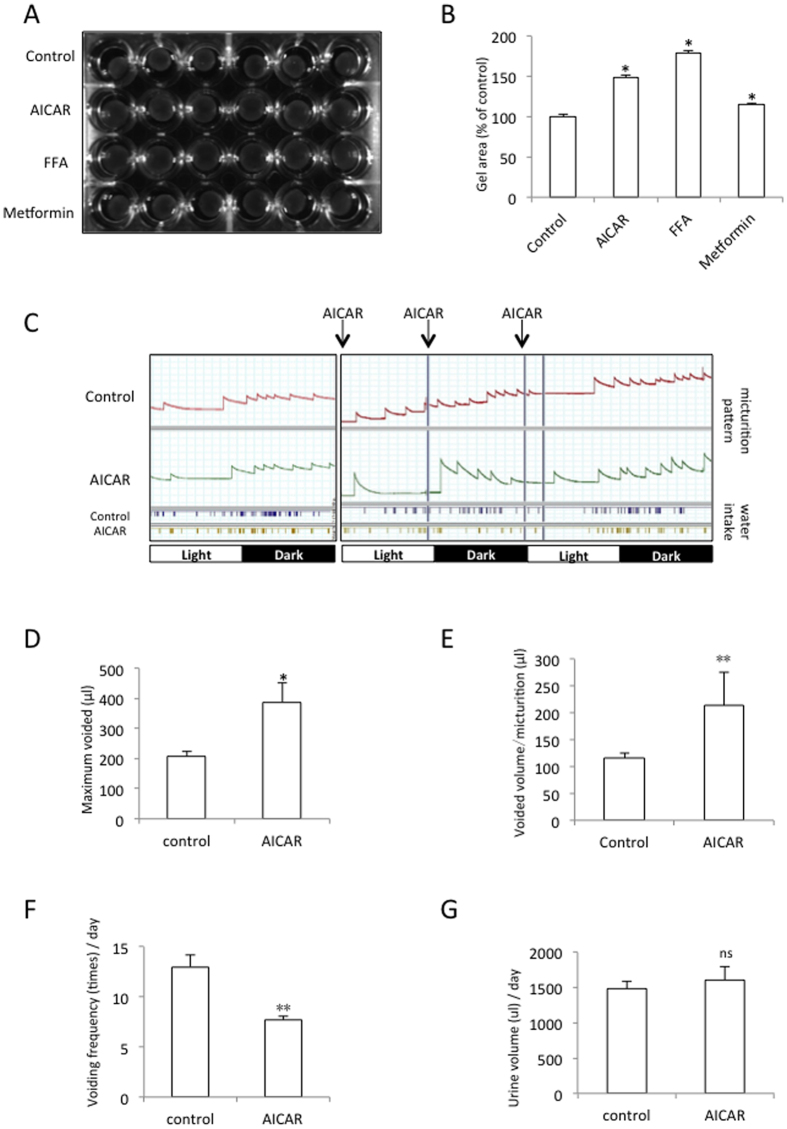
AMPK inhibits BSMC contraction and alters mouse micturition. (**A**) Effect of AMPK on cell contraction. The BSMCs were cultured in a collagen gel and stimulated with 1 mM AICAR, 50 μM FFA or 5 mM metformin for 24 h. (**B**) The gel area in (**A**) was measured using ImageJ software and is expressed as a percentage of control. The data represent the mean ± SEM of 6 independent experiments comparing the untreated cells to those stimulated with AICAR, FFA or metformin (^*^p < 0.01 versus the control, one-way ANOVA followed by Dunnett’s test). (**C**) Voiding behavior of the mice. The mice were intraperitoneally injected with AICAR (50 mg/kg) three times at 12 h intervals. The micturition patterns of the AICAR (AICAR) and sham-treated control groups (control) were recorded with the metabolic cage. On the X-axis, the black squares indicate the dark periods (9:00 pm to 9:00 am) and the white squares indicate the light periods (9:00 am to 9:00 pm). (**D**–**G**) The maximum urine volume voided per micturition (UVVM), voiding frequency and total urine volume/day in the control and AICAR-treated mice are shown. The data represent the mean ± SEM of 4 independent experiments comparing the control and AICAR-treated mice (^*^P < 0.05, ^**^P < 0.01 versus the control, two-tailed t-test).

Previous studies have demonstrated that Cx43 regulates cell contraction^22^,^39^. Therefore, we tested whether the AMPK-mediated suppression of Cx43 was associated with reduced BSMC contraction. For this purpose, the BSMCs were embedded in a collagen gel, and the cells’ contractile force in response to serum was reflected by a reduction in the surface areas of the gels^39^. As indicated in Fig. 2A,B, the addition of AMPK agonists to the BSMC-embedded gels significantly prevented the reduction in the gel area. These results indicate that AMPK suppresses BSMC contraction *in vitro*.

Given that Cx43 also affects bladder activity^9^,^23^,^40^, we therefore examined the possible influence of AMPK on mouse voiding behavior. For this purpose, we recorded and analyzed the micturition patterns of the AICAR-treated and untreated mice (Fig. 2C). The mice were intraperitoneally injected with an AICAR solution (50 mg/kg) three times at 12 h intervals. The AICAR treatment significantly increased the average and maximal urine volume voided per micturition (UVVM) (Fig. 2D,E). However, it decreased the urinary frequency (Fig. 2F). The total urine volume per day of the treated and untreated mice was not different (Fig. 2G). These results suggest that the AMPK treatment improves bladder storage capacity.

**Figure 3:**
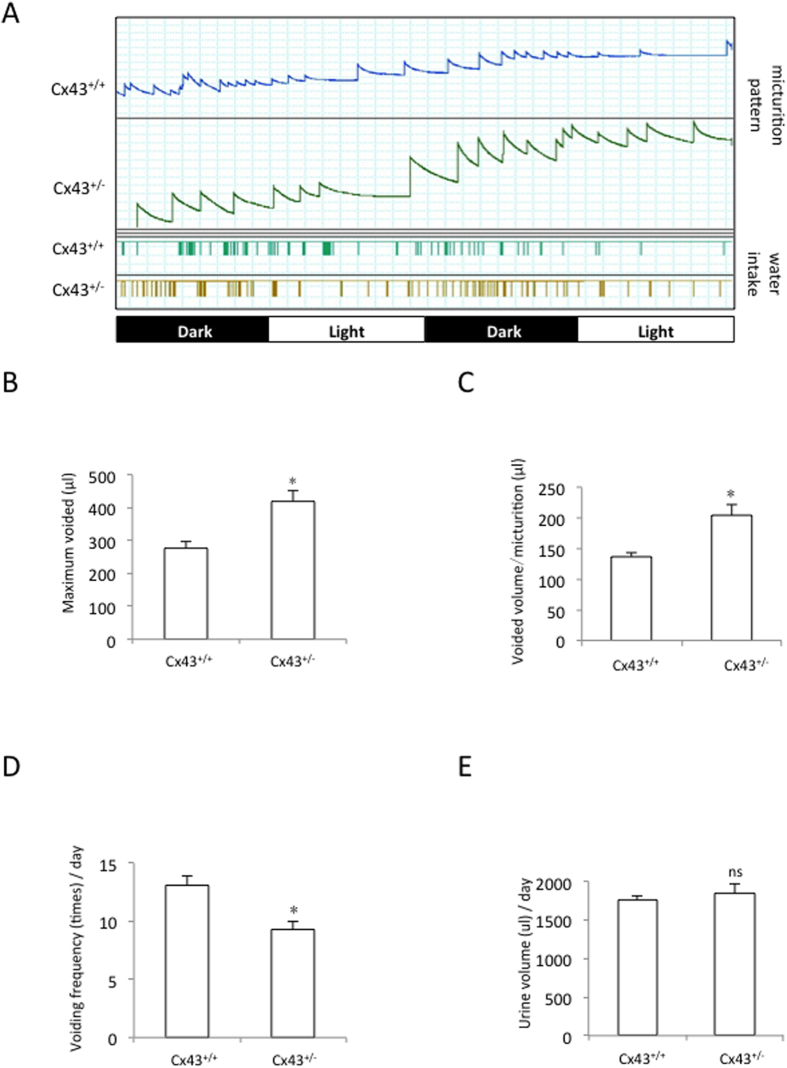
Comparison of the micturition patterns in the *Cx43*^+/+^ and *Cx43*^+/−^ mice. (**A**) The micturition patterns in the *Cx43*^+/+^ and *Cx43*^+/−^ mice were recorded as above. (**B**–**E**) The maximum UVVM, voiding frequency and urine volume/day in the Cx43^+/+^ and Cx43^+/−^ mice are shown. The data represent the mean ± SEM of 4 independent experiments comparing the Cx43^+/+^ and Cx43^+/−^ mice (^*^P < 0.01, two-tailed t-test).

To confirm that Cx43 indeed regulates mouse bladder activity, we compared the micturition patterns of *Cx43*^+/+^ and *Cx43*^+/−^ mice. The micturition patterns are shown in Fig. 3A. A quantitative analysis of the curve revealed that the *Cx43*^+/−^ mice had a higher UVVM and a lower urinary frequency than the *Cx43*^+/+^ mice (Fig. 3B–D). There was no difference in the total urine volume (Fig. 3E). These results indicate that Cx43 participates in the control of bladder capacity.

### AMPK inhibits Cx43 expression by suppressing CREB phosphorylation

**Figure 4:**
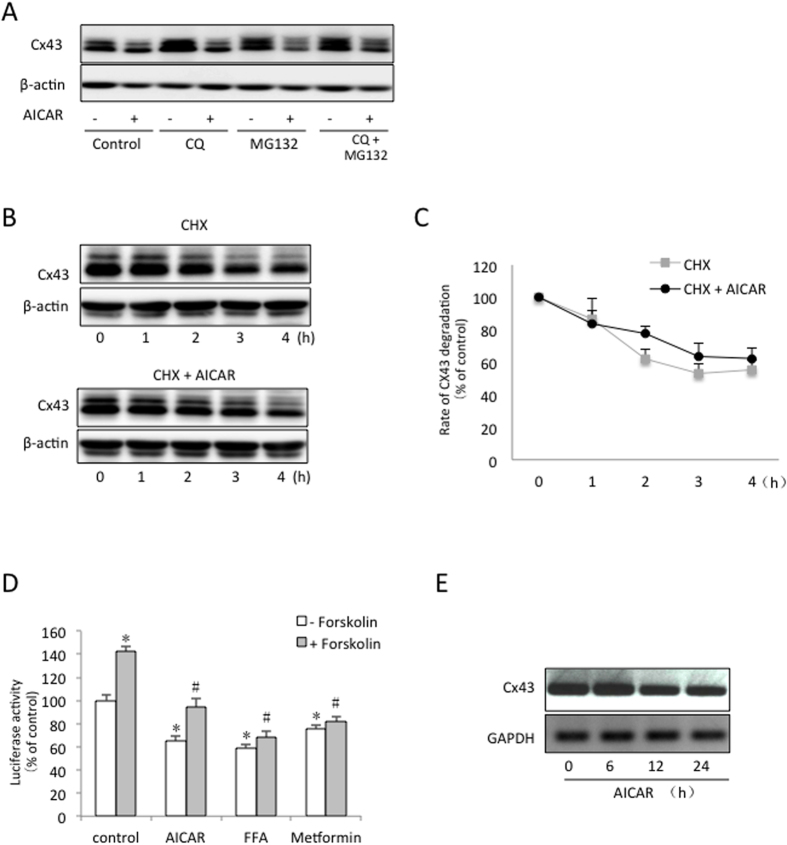
The AMPK-mediated suppression of Cx43 expression is due to its actions on protein synthesis. (**A**–**C**) Effect of AMPK on Cx43 degradation. The BSMCs were exposed to 50 μM CQ, a lysosome inhibitor, 5 μM MG132, a proteasome inhibitor, or CQ plus MG132 with or without 1 mM AICAR for 6 h. The cellular proteins were extracted and subjected to western blot analysis for Cx43. (**B**) Time-dependent effect of AICAR on Cx43 degradation. The BSMCs were exposed to 50 μg/ml cycloheximide in the presence (lower panel) or absence (upper panel) of 1 mM AICAR for the indicated times. (**C**) The intensities of the Cx43 signals in (**B**) were measured using ImageJ software and are expressed as a percentage of the zero point. The data represent the mean ± SEM of 4 independent experiments. (**D**) Effect of AMPK on Cx43 promoter activity. The BSMCs were transiently transfected with the Cx43 promoter (pCx1686-Luc) and exposed to AICAR (1 mM), FFA (50 μM), or metformin (5 mM) in the presence or absence of forskolin (10 μM) for 12 h. The relative luciferase activity is represented as the fold induction over the untreated control. The data represent the mean ± SEM of 4 independent experiments comparing the untreated cells to those stimulated with AICAR, FFA or metformin with or without forskolin (^*^P < 0.01 versus the untreated control, ^#^P < 0.05 versus forskolin alone, one-way ANOVA followed by Dunnett’s test). (**E**) Effect of AMPK on the expression of the Cx43 mRNA. The BSMCs were exposed to AICAR for the indicated periods. The total mRNA was extracted and subjected to RT-PCR analysis. GAPDH expression is shown at the bottom as a loading control.

To explore the mechanisms responsible for the reduced Cx43 expression, we examined the influence of AMPK on Cx43 turnover. We first tested whether the reduced Cx43 expression could be a consequence of accelerated Cx43 degradation. For this purpose, we blocked Cx43 degradation with lysosome and proteasome inhibitors, two major organelles that are involved in Cx43 degradation^41^, and determined whether these treatments could abolish the effect of AMPK. As expected, the cells treated with the lysosome inhibitor chloroquine (CQ) and/or the proteasome inhibitor MG132 exhibited increased basal levels of Cx43 (Fig. 4A). However, the treatments did not greatly alter the Cx43-inhibiting action of AMPK. We also assessed the influence of AMPK on the rate of Cx43 degradation. As shown in Fig. 4B,C, the inhibition of protein synthesis with cycloheximide (CHX) led to a time-dependent reduction in Cx43 expression. In the presence of AICAR, however, the rate of Cx43 degradation was not significantly affected. Thus, these results indicate that the inhibitory effect of AMPK on Cx43 was not mediated by accelerated Cx43 degradation.

We then proceeded to determine the influence of AMPK on Cx43 synthesis. Therefore, we transfected the BSMCs with a Cx43 promoter-luciferase reporter construct and conducted a promoter activity assay^21^,^22^,^42^,^43^. Figure 4D shows that AICAR, FFA and metformin significantly suppressed the luciferase activity under both basal and forskolin-stimulated conditions. Forskolin, a well-known activator of the cAMP signaling pathway, stimulated Cx43 promoter activity, as previously reported^22^,^44^,^45^. Consistent with the inhibitory effect of AMPK on the Cx43 promoter, the AMPK activator AICAR also suppressed the expression of the Cx43 mRNA (Fig. 4E). Taken together, these results indicate that AMPK suppresses Cx43 synthesis.

**Figure 5:**
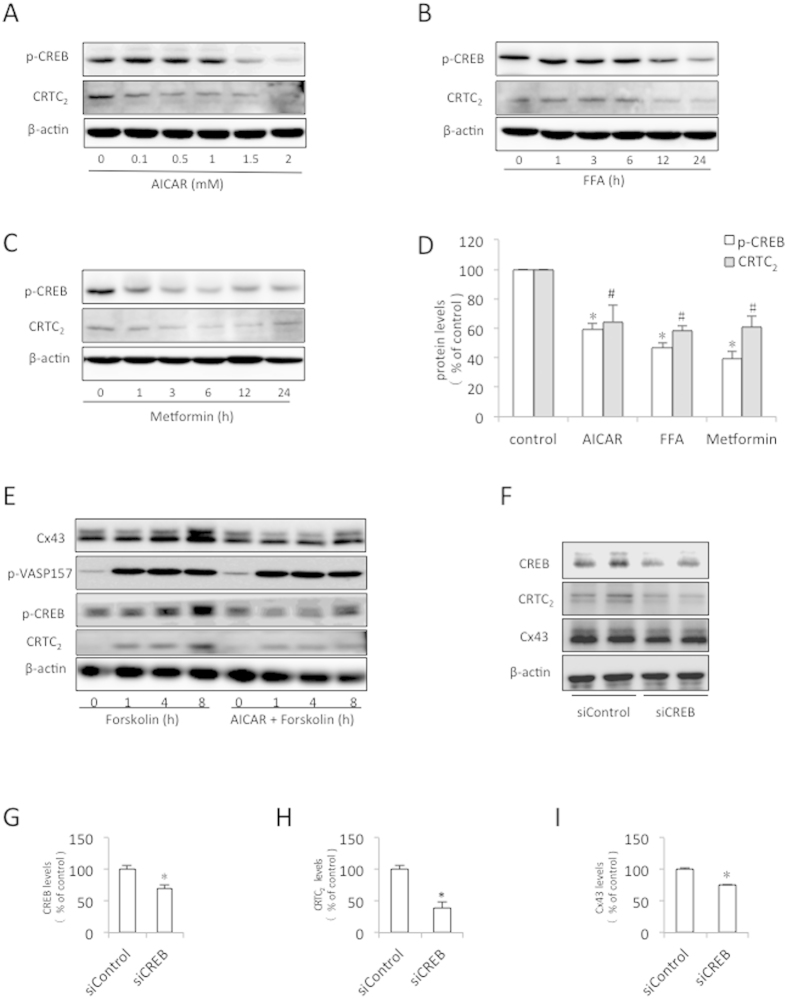
AMPK inhibits Cx43 expression by suppressing CREB phosphorylation. (**A–D**) Effect of AMPK on CREB phosphorylation and the CRTC_2_ protein level. The BSMCs were treated with various concentrations of AICAR (**A**) for 12 h, or with 50 μM FFA (**B**) or 5 mM metformin (**C**) for the indicated times. The cellular proteins were extracted and subjected to western blot analysis for CREB phosphorylation and CRTC_2_ expression. (**D**) Densitometric analysis of the p-CREB and CRTC_2_ levels after the cells were treated with the AMPK agonists. The BSMCs were exposed to 1.5 mM AICAR, 50 μM FFA, or 5 mM metformin for 12 h. The intensities of the p-CREB and CRTC_2_ signals were measured using ImageJ software. The data represent the mean ± SEM of 3 independent experiments comparing the untreated cells to those stimulated with AICAR, FFA or metformin (^*^P < 0.01 versus the control, ^#^P < 0.05 versus the control, one-way ANOVA followed by Dunnett’s test). (**E**) Effect of AMPK on the forskolin-induced changes in several signaling molecules. The BSMCs were pretreated with 1 mM AICAR for 1 h and then exposed to 10 μM forskolin for the indicated times. The cellular proteins were subjected to Western blot analysis for Cx43, phosphorylated VASP157, phosphor-CREB and CRTC_2_. (**F**) The Cx43 protein levels were inhibited with a specific siRNA against CREB. The BSMCs were transfected with a CREB siRNA or control siRNA for 48 h. The cellular proteins were subjected to Western blot analysis for Cx43 and CREB. (**G**–**I**) Quantitative analysis of the CREB and Cx43 levels shown in (**F**). The results are expressed as the fold induction relative to the basal levels. The data represent the mean ± SEM of 3 independent experiments comparing the siControl- and siCREB-transfected cells (^*^P < 0.05, two-tailed t-test).

To elucidate the mechanism by which AMPK inhibited Cx43 promoter activity, we focused on CREB, a well-characterized transcriptional factor involved in the activation of the Cx43 gene^10^,^22^,^45^. Figure 5A–D show that the AMPK activators inhibited CREB phosphorylation in a time- and concentration-dependent fashion. They also concomitantly decreased the protein level of the CREB coactivator CRTC_2_. The activation of the cAMP signaling pathway by forskolin resulted in increased phosphorylation of VASP and CREB (two substrates of PKA), and induced the expression of CRTC_2_ and Cx43. In the presence of AICAR, the effects of forskolin on CREB, CRTC_2_ and Cx43 were markedly suppressed (Fig. 5E). Intriguingly, AICAR did not influence the level of VASP phosphorylation, suggesting that AMPK interfered with CREB phosphorylation through a PKA-independent mechanism.

To determine the role of CREB in modulating Cx43 expression, we knocked down CREB with a specific siRNA. CREB downregulation reduced Cx43 and CRTC_2_ expression (Fig. 5F–I). Together, these observations indicate that AMPK inhibits Cx43 by suppressing CREB.

### AMPK suppresses the pro-inflammatory cytokine-induced Cx43 expression and function in BSMCs

**Figure 6:**
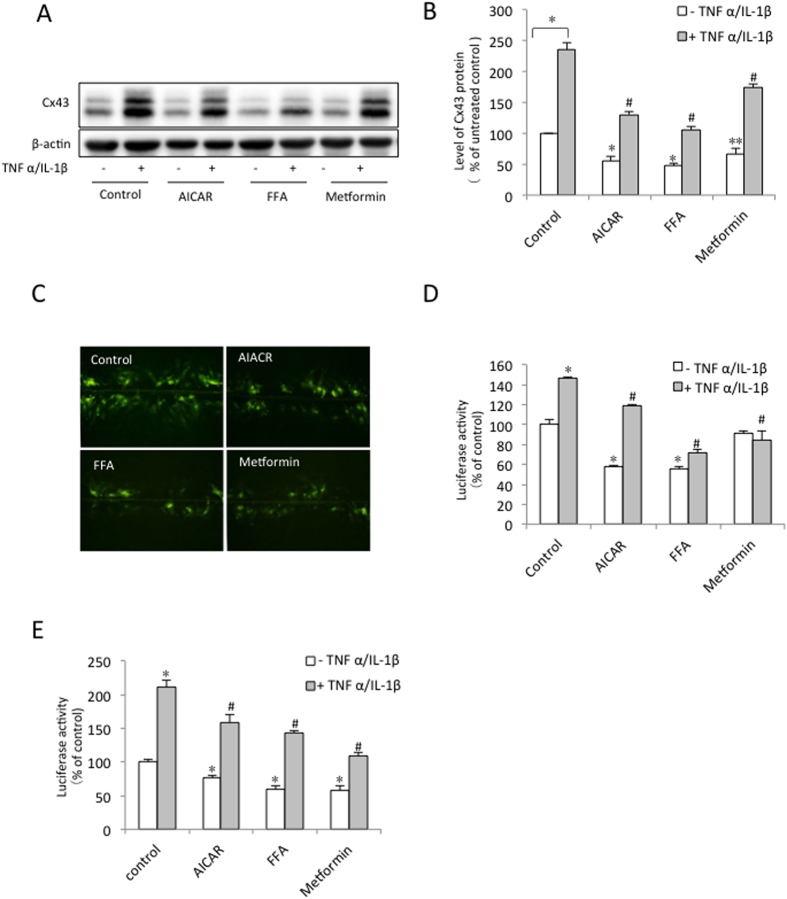
AMPK suppresses TNFα/IL-1β-induced Cx43 expression and function. (**A**) Effect of the AMPK agonists on TNFα/IL-1β-induced Cx43 expression. The BSMCs were exposed to 20 ng/ml TNFα plus 2 ng/ml IL-1β with or without 1 mM AICAR, 50 μM FFA or 5 mM metformin for 24 h. (**B**) Densitometric analysis of the Cx43 expression shown in (**A**). The data represent the mean ± SEM of 4 independent experiments (^*^P < 0.01. ^**^P < 0.05 versus the untreated control, ^#^p < 0.01 versus the cytokines alone, one-way ANOVA followed by Dunnett’s test). (**C**) Effect of AMPK on TNFα/IL-1β-induced gap junctional intercellular communication. The BSMCs were pretreated with 1 mM AICAR, 50 μM FFA, or 5 mM metformin for 1 h, and then exposed to 20 ng/ml TNFα and 2 ng/ml IL-1β. The micrographs of LY (green) diffusion into cellular monolayer after scrape loading are shown (magnification, ×200). (**D**) Effect of AMPK on Cx43 promoter activity. The BSMCs were transiently transfected with the Cx43 promoter construct (pCx1686-Luc) and then exposed to AICAR (1 mM), FFA (50 nM), or metformin (5 mM) in the presence or absence of TNFα (20 ng/ml)/IL-1β (2 ng/ml) for 12 h. The luciferase activity is represented as the fold induction over untreated control. The data represent the mean ± SEM of 4 independent experiments (^*^P < 0.05 versus the untreated control, ^#^P < 0.01 versus the cytokines alone, one-way ANOVA followed by Dunnett’s test). (**E**) Effect of AMPK on NF-κB promoter activity. The BSMCs were transiently transfected with the NF-κB promoter construct and exposed to AICAR (1 mM), FFA (50 μM), or metformin (5 mM) in the presence or absence of TNFα (20 ng/ml)/IL-1β (2 ng/ml) for 10 h. The relative luciferase activity is represented as the fold induction over the untreated control. The data represent the mean ± SEM of 4 independent experiments (^*^P < 0.01 versus the untreated control, ^#^P < 0.01 versus the cytokines alone, one-way ANOVA followed by Dunnett’s test).

To determine whether AMPK also inhibits Cx43 expression under pathological conditions, we examined the influence of AMPK on the inflammatory mediator-induced increase in Cx43 expression^22^. The BSMCs incubated with TNFα/IL-1β displayed pronounced elevation in Cx43 and GJIC expression. In the presence of AMPK agonists, however, this effect was significantly blunted (Fig. 6A–C). The AMPK agonists also significantly repressed the cytokine-induced activation of the Cx43 promoter (Fig. 6D). Given that our previous studies revealed that the TNFα/IL-1β-induced increase in Cx43 expression was mediated by NF-κB activation^22^, we also examined the effect of AMPK on NF-κB promoter activity. As shown in Fig. 6E, the AMPK agonists significantly suppressed TNFα/IL-1β-triggered NF-κB activation.

### AMPK attenuates cyclophosphamide (CYP)-induced cystitis

**Figure 7:**
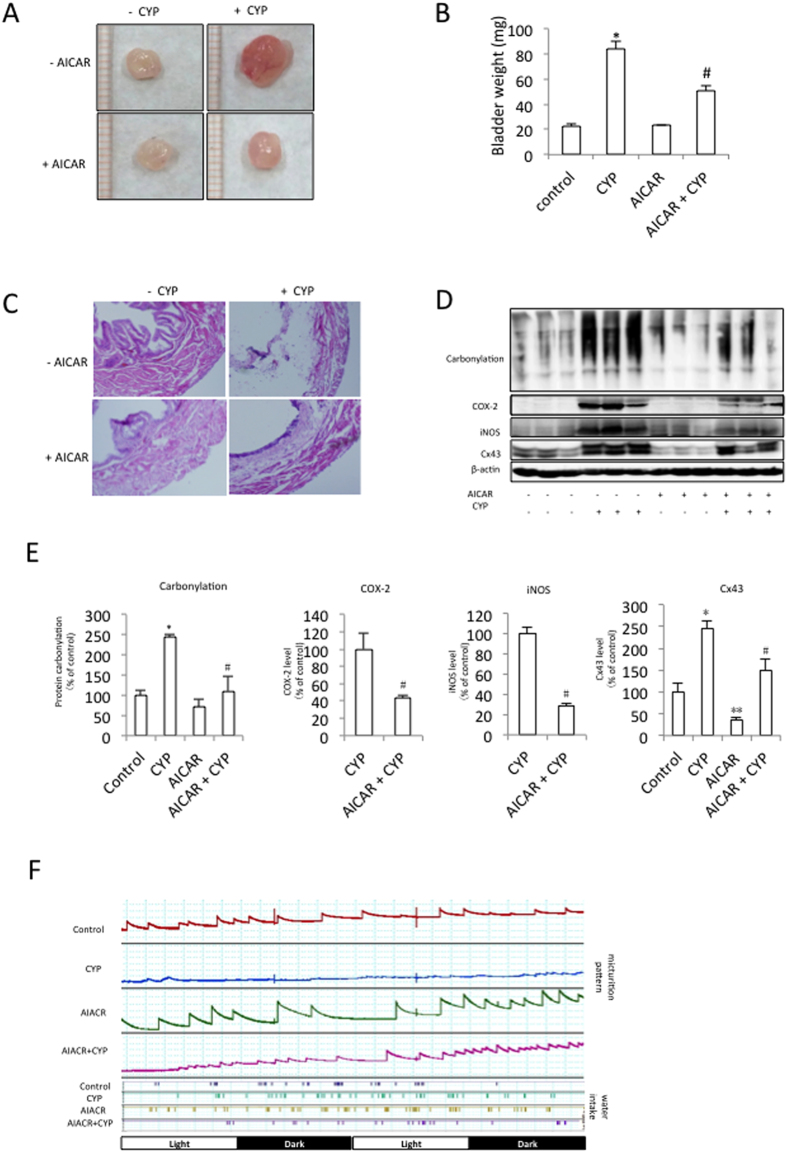
AMPK attenuates CYP-induced cystitis. (**A**) Effect of AICAR on CYP-induced cystitis. The mice were divided into 4 groups: control, AICAR-treated control, CYP, and CYP and AICAR. The mice were intraperitoneally injected with 200 mg/kg AICAR three times at 12-hour intervals. CYP (300 mg/kg) was injected 2 h after the last AICAR injection, and the bladder morphology, weight, histochemistry and bladder protein extractions were performed after 24 h. The control mice were injected with saline. (**A**) Representative images of the mouse bladder sections from the control and AICAR-treated groups. Note the obvious congestion, enlargement and hemorrhaging in the CYP-treated bladder. (**B**) Bladder weight in the different groups. The data represent the mean ± SEM of 3 independent experiments and 3 randomly chosen mice per group (*P < 0.01 versus the control; ^#^P < 0.05 versus the CYP-treated group, two-way ANOVA followed by Bonferroni’s test). (**C**) Representative histological changes in the bladder. Images of the hematoxylin-eosin-stained bladder sections (magnification, ×40). (**D**) The bladder tissue proteins were extracted and subjected to Western blot analysis for protein carbonylation, COX-2, iNOS, Cx43 and β-actin. (**E**) Quantitative analysis of the protein levels shown in (**D**). The results are expressed as the fold induction relative to the control. The data presented in A to D are one representative experiment (n = 3–4 mice/group) out of 3 separate studies with similar results. (^*^P < 0.01, ^**^P < 0.05 versus the untreated control; ^#^P < 0.05 versus the CYP-treated group, two-way ANOVA followed by Bonferroni’s test). (**F**) The micturition patterns of the different groups were shown. The mice were treated as described above. The micturition patterns were recorded after CYP injection. On the X-axis, the black squares indicate the dark periods (9:00 pm to 9:00 am) and the white squares indicate the light periods (9:00 am to 9:00 pm). Representative data from four separate experiments (n = 4) with similar results are shown.

To determine whether the Cx43-inhibiting action of AMPK could be exploited to treat voiding dysfunction, we examined the effect of AMPK activation on CYP-induced mouse cystitis^23^,^46^. The mice were intraperitoneally injected with an AICAR solution (50 mg/kg) three times at 12 h intervals before CYP administration. The intraperitoneal injection of CYP (300 mg/kg) into the mice resulted in bladder hemorrhages, congestion and edema (Fig. 7A). The weight of the CYP-treated mouse bladder was four times heavier than that of the untreated control (Fig. 7B). AICAR-mediated AMPK activation greatly attenuated the bladder congestion and edema (Fig. 7A,B). The CYP-treated mice exhibited clear signs of lamina propria edema in bladder sections (Fig. 7C). This phenotype was markedly reduced in the presence of AICAR (Fig. 7C).

The Western blot analysis revealed that CYP induced protein oxidation and iNOS and COX_2_ expression, which were also significantly attenuated by AICAR (Fig. 7D,E). Given that Cx43 is a key regulator of bladder function^14^,^17^,^18^,^19^, we examined the changes in Cx43 expression in the model mice. As shown in Fig. 7D,E, CYP administration significantly elevated Cx43 expression in the bladder. This action of CYP was largely blocked by AICAR. AICAR also significantly suppressed the basal level of Cx43 expression in the bladder. Of note, the mice that were treated with another AMPK agonist, metformin, exhibited the similar results (Supplementary Figure 1).

A functional analysis revealed that AMPK attenuated CYP-induced mouse bladder dysfunction. As shown in Fig. 7F, the mice injected with CYP exhibited a severe alteration in bladder function, as manifested by the markedly increased micturition frequency and decreased micturition volume. In stark contrast, the mice treated with AICAR retained a relatively normal micturition pattern. Because of the extremely high frequency and low volume, the micturition curve for the CYP-treated mice was almost a straight line. A quantitative analysis of the micturition was not performed. Taken together, these results indicate that AMPK suppresses CYP-induced cystitis in mice.

### The *Cx43* ^+/−^ mouse displays a relatively normal urination pattern in response to CYP compared to the *Cx43* ^+/+^ mouse

**Figure 8:**
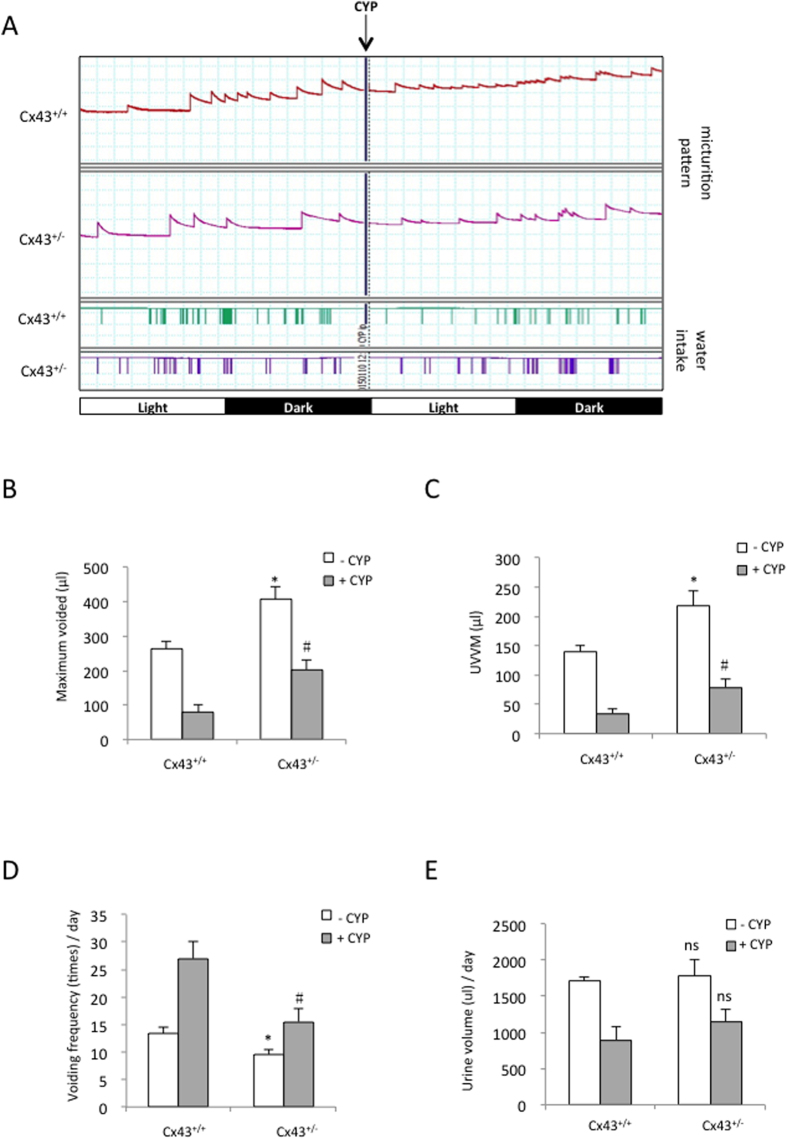
The heterozygous Cx43 mouse displays a relatively normal micturition pattern in response to CYP. (**A**) Micturition patterns in *Cx43*^+/+^ and *Cx43*^+/−^ mice before and after the intraperitoneal injection of CYP. The micturition patterns of the *Cx43*^+/+^ and *Cx43*^+/−^ mice before and after an intraperitoneal injection of 100 mg/kg CYP were recorded with the metabolic cage. (**B**–**D**) The maximum voided volume, UVVM, voiding frequency and total urine volume/day in the *Cx43*^+/+^ and *Cx43*^+/−^ mice in response to CYP are shown. The data represent the mean ± SEM of 3 independent experiments (^*^P < 0.01 versus the untreated *Cx43*^+/+^ control; ^#^P < 0.05 versus the CYP-treated *Cx43*^+/+^ mice, two-tailed t-test).

To determine whether the difference in Cx43 expression was sufficient to affect CYP-induced micturition dysfunction, we compared micturition patterns of the *Cx43*^+/+^ and *Cx43*^+/−^ mice following an intraperitoneal injection of CYP (Fig. 8A). In our initial experiment, CYP was used at a relatively high concentration (300 mg/kg). We failed to detect an obvious difference in the micturition patterns of the *Cx43*^+/+^ and *Cx43*^+/−^ mice (data not shown). The micturition curve was almost a straight line. However, when CYP was used at a lower concentration (100 mg/kg), the *Cx43*^+/−^ mice displayed a relatively normal urination pattern and had a significantly higher UVVM and lower voiding frequency than the *Cx43*^+/+^ mouse (Fig. 8B–E). Intriguingly, under these conditions, there were no obvious differences in bladder protein oxidation and the expression of iNOS and COX_2_ between the CYP-treated *Cx43*^+/+^ and *Cx43*^+/−^ mice (data not shown).

## Discussion

In this study, we demonstrated that AMPK inhibited Cx43 expression and function in BSMCs. Furthermore, we showed that AMPK weakened BSMC contraction and altered bladder activity under both normal and pathological conditions. Our study thus characterizes AMPK as an inhibitor of Cx43 channels and suggests that AMPK can be used to treat bladder hyperactivity.

AMPK has emerged as a key regulator of ion channels, and regulates multiple channel activities^27^. In this study, we characterized AMPK as an inhibitor of GJs. Three structurally and functionally different AMPK agonists, AICAR, FFA and metformin, all suppressed Cx43 expression and function in BSMCs. Given that these agonists activate AMPK through different signaling pathways (AICAR alters the intracellular AMP concentrations; metformin increases the ability of LKB1 to phosphorylate AMPK; and FFA activates the Ca^2+^-CaMKKβ pathway)^32^,^33^,^47^, it is conceivable that their common AMPK-activating action mediated the suppressive effect. In accord with this notion, AMPK inhibition indeed increased Cx43 expression.

The suppressive effect of AMPK on Cx43 expression was mediated by the inhibition of CREB, a transcription factor that binds to cAMP response element sites and regulates diverse cellular responses. Previous studies have confirmed the presence of CREB-binding sites in the Cx43 gene. Furthermore, it has been extensively demonstrated that the cAMP signaling pathway stimulates Cx43 expression and function^22^,^39^. Currently, the mechanisms by which AMPK inhibited CREB are unclear. Lee *et al*. reported that the activation of AMPK with AICAR or metformin repressed hepatic gluconeogenesis by disrupting the CREB-CRTC_2_ complex with its orphan nuclear receptor small heterodimer partner^48^. The same mechanism may operate in the current pathway. Intriguingly, AMPK also suppressed the level of the CRTC_2_ protein, a CREB-regulated transcriptional factor that facilitates the binding of CREB to cAMP response elements^48^,^49^,^50^. Several previous studies show that AMPK blocks the transcriptional function of CRTC_2_ by promoting CRTC_2_ phosphorylation and restricting it to the cytosol^48^,^50^,^51^. In this study, we observed that downregulation of CREB with siRNA also resulted in a reduction in CRTC_2_ expression, indicating that AMPK also indirectly regulates CRTC_2_ expression by suppressing CREB. Thus, the disruption of the CREB-CRTC_2_ complex could be an important mechanism by which AMPK regulates Cx43 expression.

Previous studies from our and other groups have shown that Cx43 plays an important role in regulating cell contraction, presumably by synchronizing and integrating the intercellular calcium signals, which are required for coordinated cell contraction. The disruption of GJs or knockdown of Cx43 resulted in reduced cell contraction^18^,^21^,^39^. A comparison of the fibroblasts derived from mice expressing different amounts of Cx43 revealed that cells deficient in or expressing lower levels of Cx43 (*Cx43*^*−/−*^ and *Cx43*^+/−^ mice) exhibited weaker contractile responses to stimuli in the gel contraction assay compared to the Cx43^+/+^ cells^21^. Clinical and animal studies also implicate Cx43 in the control of detrusor activity and bladder function. A recent study by Negoro *et al*. showed that the circadian oscillation of connexin43 expression in BSMCs contributes to the diurnal changes in bladder capacity^9^. Consistent with these findings, Huang *et al*. reported that a genetically modified Cx43 mouse displayed mutation-specific changes in bladder function. The Cx43 (I130T) mice exhibited reduced urination frequency^40^. The BSMCs from these mice had a reduced level of Cx43 expression and a weak contractile response in collagen gel. In agreement with these reports, we also observed that the Cx43 heterozygous mice (*Cx43*^+/−^) had a higher bladder storage capacity than the *Cx43*^+/+^ mice. Furthermore, they retained a much better micturition pattern than the *Cx43*^+/+^ mice in CYP-induced cystitis. In this context, it is reasonable to speculate that the effect of AMPK on BSMC contraction and micturition was most likely mediated by its inhibitory action on Cx43 expression.

AMPK may bring additional benefits to the afflicted bladder through its anti-oxidative and anti-inflammatory effects. Oxidative stress and inflammation are two major pathological changes in a variety of bladder disorders^52^,^53^,^54^. In CYP-induced cystitis, the metabolic product of CYP, acrolein, accumulates in the bladder, where it evokes oxidative stress, inflammatory responses and urothelial cell injury^53^,^55^,^56^. AMPK has strong antioxidant actions. It reduces ROS production and enhances the antioxidant system through multiple mechanisms^29^,^57^,^58^,^59^. Moreover, AMPK also has anti-inflammatory properties^32^,^33^. It inhibits cytokine-induced activation of NF-κB and the production of inflammatory mediators^32^,^33^. Because oxidative stress and inflammation are implicated in a variety of bladder disorders, AMPK could be applicable to many bladder situations. It is especially true for the case of inflammation-associated urinary frequency. We have reported that the inflammatory mediators NO and prostaglandin potently induced bladder Cx43 expression by activating the cAMP signaling pathway^22^,^42^,^45^. AMPK could block their effects through multiple mechanisms, including suppressing their production by inhibiting NF-kB activation and intercepting their Cx43-elevating action by interfering with CREB phosphorylation. AMPK might also be beneficial for the prevention and treatment of metabolic syndrome-associated bladder alterations by improving the metabolic status^26^,^35^,^36^.

Collectively, our results indicate that AMPK suppresses Cx43 expression and function in BSMCs by inhibiting CREB. The *in vivo* activation of AMPK increases the bladder storage capacity under normal conditions and ameliorates voiding dysfunction in a mouse model of cystitis. Thus, our study suggests that strategies targeting AMPK can be developed as a novel therapeutic approach for treating bladder dysfunction.

## Materials and Methods

### Reagents

Anti-phospho-AMPKα (Thr172; #07-681) and OxyBlot™ Protein Oxidation Detection Kit were purchased from Merck Millipore (EMD Millipore, Billerica, MA). Cox-2 antibody (160107) and 5-aminoimidazole-4-carboxamide ribonucleoside (AICAR) were purchased from Cayman Chemical (Ann Arbor, MI). Anti-iNOS (ADI-KAS-NO 001) was obtained from Enzo Life Sciences (NY, USA). Anti-β-actin antibody (#4970) and horseradish peroxidase–conjugated anti-rabbit IgG (#7074) were obtained from Cell Signaling Technology (Danvers, MA). Metformin was obtained from Wako (Osaka, Japan). IL-1β and TNFα were purchased from R&D Systems (Minneapolis, MN). Anti-Cx43 (c6219), cyclophosphamide (CYP), flufenamic acid (FFA; 2-[3-(trifluoromethyl)phenylamino] benzoic acid), Fetal bovine serum (FBS), Chloroquine (CQ), MG132, cycloheximide (CHX), trypsin/EDTA, antibiotics, and all other chemicals were purchased from Sigma (Tokyo, Japan).

### Cells

Primarily cultured BSMCs were obtained from the bladders of Sprague–Dawley rats, as we have previously reported^60^. Briefly, the bladder body was placed on ice, and the outer layers (tunica serosa and tunica adventitia) and the inner layers (tunica intima and the epithelium) were removed. The remaining tunica media was also removed with a cotton swab. The smooth muscle layer was then incubated with shaking at 37 °C for 30min in phosphate-buffered saline containing 0.2% trypsin. After the incubation, the tissue was minced and suspended in Dulbecco’s modified Eagle’s medium nutrient mixture F-12 (DMEM/ F-12; GIBCO-BRL, Gaithersburg, MD, USA) supplemented with 0.1% collagenase using Pasteur pipettes. The suspension was further incubated at 37 °C for 30 min and centrifuged for 5 min. The pellet was resuspended in DMEM/F-12 containing 10% fetal bovine serum (FBS; Sigma-Aldrich) and centrifuged at 250 G for 2 min, and the supernatant fraction containing BSMCs was used for culture. For maintenance, cells were cultured with DMEF/F12 supplemented with 10% FBS and 1% Antibiotic Antimycotic Solution (sigma, A5955) in a humidified atmosphere of 5% CO_2_/95% air at 37 °C. For experiment, BSMCs were incubated in complete medium with 1% FBS. The cells used in this investigation exhibited an elongated spindle-like morphology. Immunofluorescent staining of α-smooth muscle actin, a differentiation marker of SMCs, revealed that more than 95% of cells were strongly positive^60^.

### Animals

Adult female Cx43 wild-type (*Cx43*^+/+^) and heterozygous Cx43 knockout mice (*Cx43*^+/−^) weighing 20 to 25 g were bred from the offspring of *Cx43*^+/+^ or *Cx43*^+/+^ mice mated with heterozygous Cx43 knockout mice (B6;129-Gja1<tm1Kdr>/J; Jackson Laboratories, Bar Harbor, ME, USA). The mice were housed in the containment facilities of the Animal Center and fed food and water in an air-conditioned room with a 12-h light/dark cycle. The genotypes of all mice were determined by polymerase chain reaction (PCR), according to the protocol provided by Jackson Laboratories. All animal experiments were approved by the animal experiment committee of the University of Yamanashi and performed in accordance with the relevant guidelines and regulations.

### CYP-Induced Mouse Cystitis

To induced mouse cystitis, the adult female wild-type (*Cx43*^+/+^) and heterozygous Cx43 (*Cx43*^+/−^) mice were intraperitoneally injected with 300 mg/kg or 100 mg/kg CYP, as indicated in the Figure Legend. The control mice received the same volume of saline.

### Western Blot Analysis

The isolated bladders were homogenized in lysis buffer (8 mol/L urea, 1 mmol/L dithiothreitol, 1 mmol/L ethylenediaminetetraacetic acid, 50 mmol/L Tris-HCl, pH 8.0) on ice (1000 μl lysis buffer per bladder). Lysates were incubated on ice for 30 min with intermittent mixing and then sonicated for 15 seconds two times. After that samples were centrifuged at 12,000 rpm for 30 min at 4 °C, supernatant was recovered and protein concentration was determined using the Pierce Micro BCA Protein Assay Kit (Thermo Fisher Scientific, Waltham, MA). All the samples were diluted to a level of 2% SDS by adding 5× sample buffer (7.55% Tris, 50% Glycerol, 10% SDS) in a ratio of 4:1 (4 vol sample: 1 vol 5× buffer). Cell proteins extraction and western blot analysis were performed as described previously^25^,^34^,^61^. Briefly, extracted proteins were loaded onto 10% or 4–20% SDS-polyacrylamide gels and electrotransferred onto polyvinylidene difluoride membranes. After blocking with 5% non-fat dry milk in PBS, the membranes were incubated with primary antibody overnight at 4 °C. After washing, the membranes were probed with horseradish peroxidase-conjugated anti-rabbit or anti-mouse IgG, and the bands were visualized using Chemi-Lumi One L (Nacalai Tesque, Kyoto, Japan). The chemiluminescent signal was captured with a Fujifilm luminescent image LAS-1000 analyzer (Fujifilm, Tokyo, Japan). The results were quantified using Image J software. β-actin was used as internal control.

### Immunocytochemical Analysis

The cells were fixed with 3% paraformaldehyde in PBS for 10 min and permeabilized with 0.5% Triton X-100 for 15 min. After washing with PBS, 0.05% tween-20, cells were incubated overnight with anti-Cx43 antibody (diluted 1:100 in 1% BSA in PBS; 4 °C). After rinsing with PBS, cells were incubated with fluorochrome-conjugated secondary antibodies (diluted 1:200 in 1% BSA in PBS; 37 °C) for 1 to 2 h. Afterward, cells were washed with PBS and stained with DAPI for 10 min before final washing. The slides were covered with Tris-buffered Mowiol (pH 8.6), and microscopy was performed with an Olympus BX50 microscope with a 40× Planapo and 570-nm emission filter. Immunofluorescence images were captured using a CCD camera attached to the microscope.

### Scrape Loading Dye Transfer

The SLDT assay was used to assess GJIC and performed as reported^22^. Cells were exposed to stimuli for 12 h before exposed to 0.05% Lucifer Yellow (diluted in PBS). A scrape line on the monolayer was made with a surgical blade and then incubated for 3 min. After washing out the background fluorescence with 1% FBS DMEM/F-12, the cells were fixed with 3% paraformaldehyde and dye transfer results were examined using an Olympus BX50 microscope with a 40× Planapo and FITC (green) filter and images were photographed using a CCD camera attached to the microscope.

### Promoter Assay

BSMCs were transfected with NF-kβ vector (Panomics, Fremont, CA) or pCx43-luciferase vector (1686-Luc, which was kindly gifted by Dr. Stephen J. Lye from Samuel Lunenfeld Research Institute, University of Toronto, Ontario, Canada) using Gene Juice according to the manufacturer’s instructions (Novagen). After 48 h, the transfected cells were exposed to different stimuli in fresh D/F-12 medium containing 1% FBS for another 10 h. After that, the cells were harvested with reporter lysis buffer. Luciferase activity of the lysate was measured with luciferin reagent, following the manufacturer’s protocol. The lysates were subjected to assays using a luminometer (Gene Light 55; Microtech Nition, Chiba, Japan). All assays were performed in quadruplicate.

### RT-PCR

The total RNA was extracted from the BSMCs using a fastPure^TM^ RNA Kit (TaKaRa, Shiga, Japan). The first-strand cDNA was synthesized using a Prime-Script RT reagent Kit (TaKaRa, Ohtsu, Japan). PCR were performed using a kit from TaKaRa (Ohtsu, Japan) and optimized according to the protocol provided by manufacturer. The Cx43 and GADPH primers used for PCR were custom synthesized by the Gibco BRL Co. and Takara Co. (Tokyo, Japan).

### Transient Transfection of the Cells with an siRNA

The BSMCs were transiently transfected with siRNAs that specifically targeted the genes or a negative control siRNA (AllStars Negative Control siRNA; QIAGEN, Tokyo, Japan) at a final concentration of 10 nM using the HiPerFect transfection reagent for 24 h. After transfection, the cellular proteins were extracted and subjected to a Western blot analysis for target proteins.

### Collagen Gel Contraction Assay

The gel contraction assays were performed as described by Yao *et al*.^39^. Briefly, 1 × 10^5^/ml BSMCs in Dulbecco’s modified Eagle’s medium were mixed with a collagen solution and incubated in 24-well plates at 37 °C. After gel formation, the cells were exposed to AICAR, FFA or metformin. The images of the gels were captured with a Fujifilm luminescent image LAS-1000 analyzer (Fujifilm, Tokyo, Japan). The surface area was measured using ImageJ software.

### Assessment of Protein Oxidation

The oxidative modification of the proteins was analyzed using the OxyBlot Protein Oxidation Detection Kit (EMD Millipore, Billerica, MA) according to the manufacturer’s instructions. Briefly, the protein lysate was prepared by suspending the mouse bladder tissue in lysis buffer (8 mol/L urea, 1 mmol/L dithiothreitol, 1 mmol/L ethylenediaminetetraacetic acid, and 50 mmol/L Tris-HCl, pH 8.0) containing a proteinase inhibitor cocktail (Nacalai Tesque, Kyoto, Japan) and 50 mM DTT. We transferred 5 μl protein samples into Eppendorf tubes with a final protein concentration of 15 μg to 20 μg. Then, 5 μl of 12% SDS and 10 μl of a 1 × DNPH (2,4-dinitrophenylhydrazine) solution were added to denature and derivatize the proteins, respectively. After 15 min incubation at room temperature, 7.5 μl of neutralization solution were added to each tube. Then, the samples were subjected to Western blot analysis.

### Histochemistry Assay

The mouse bladder tissues were fixed with 10% paraformaldehyde overnight at room temperature, followed by 30% sucrose for an additional 24 hr. The bladder tissues were then embedded in Tissue-TEK OCT compound (Sakura, Torrance, CA, USA), frozen in liquid nitrogen, and 5 μm thick sections were cut with a microtome. The tissue sections were stained with hematoxylin and eosin (H&E) for tissue histology.

### Micturition Analysis of the Mice

The metabolic cage system was performed according to the manufacturer’s instructions to evaluate the animals’ voiding behavior. The mice were housed in metabolic cages in a soundproof room at 25 °C on a 12-h light and 12-h dark cycle and provided free access to food and water. After the mice had adapted to the cage environment for 2 days, Micturition was monitored by recording the weight of the voided urine using a container that was placed under the cage and rested on a microbalance, which was connected to a computer. The system also allowed us to record the volume of water consumed. The collected data were analyzed using the Powerlab LabChart software (AD Instruments).

### Statistical Analysis

All results are expressed as the mean ± SEM. The differences between two groups were compared with the Student’s t-test. The differences between multiple groups were compared with ANOVA, followed by Dunnett’s test or Bonferroni’s test. All of the analyses were performed using SigmaPlot (Systat Software). P < 0.05 was considered statistically significant.

## Additional Information

**How to cite this article**: Zhang, X. *et al*. AMPK Suppresses Connexin43 Expression in the Bladder and Ameliorates Voiding Dysfunction in Cyclophosphamide-induced Mouse Cystitis. *Sci. Rep.* **6**, 19708; doi: 10.1038/srep19708 (2016).

## Supplementary Material

Supplementary Information
