# Supplementary material for: AMPK Suppresses Connexin43 Expression in the Bladder and Ameliorates Voiding Dysfunction in Cyclophosphamide-induced Mouse Cystitis
Source: Sci Rep. 2016 Jan 25;6:19708. doi: 10.1038/srep19708 (PMC4726257; doi:10.1038/srep19708)

AMPK Suppresses Connexin43 Expression in the Bladder and Ameliorates Voiding Dysfunction in Cyclophosphamide-induced Mouse Cystitis

Xiling Zhang[1, 4], Jian Yao[1*], Kun Gao[1], Yuan Chi[1], Takahiko Mitsui[2], Tatsuya Ihara[2], Norifumi Sawada[2], Manabu Kamiyama[2], Jianglin Fan[3], Masayuki Takeda[2]

[1] Department of Molecular Signaling, [2]Department of Urology, [3]Department of Molecular Pathology, Interdisciplinary Graduate School of Medicine and Engineering, University of Yamanashi, Yamanashi, Japan

[4] Department of Urology, The 4th affiliated hospital of China Medical University, Shenyang, China

*Correspondence: Dr. Jian Yao, Department of Molecular Signaling, Interdisciplinary Graduate School of Medicine and Engineering, University of Yamanashi, Chuo, Yamanashi 409-3898, Japan.

Tel/Fax: +81-55-273-8074 E-mail: yao@yamanashi.ac.jp

Running title: AMPK regulates bladder Cx43 and function

Key words: AMP-activated protein kinase; Connexin 43; cAMP response element-binding protein (CREB); mouse micturition dysfunction; bladder smooth muscle cell contraction.

Supplementary Figure 1. Metformin improves CYP-induced cystitis. (A) Effect of metformin on CYP-induced cystitis. Mice were divided into 4 groups: control, CYP control, metformin control and metformin plus CYP. Mice were freely accessed to drinking water containing 2 mg/ml metformin for 7 days before intraperitoneal injection of 300 mg/kg CYP. After 24 h, bladders were taken out and analyzed for pathological changes. (A) Representative image of mouse bladder from control and metformin-treated groups. (B) Bladder tissue proteins were extracted and subjected to Western blot analysis for protein carbonylation, Cox-2, iNOS, Cx43 and β-actin. (C-E) Quantitative analysis of protein levels shown in (B). Results are expressed as induction relative to the basal level (mean ± SEM, n = 3). $^{*}P < 0.01$, $^{**}P < 0.05$ versus control; $^{\#}p < 0.05$ versus CYP.

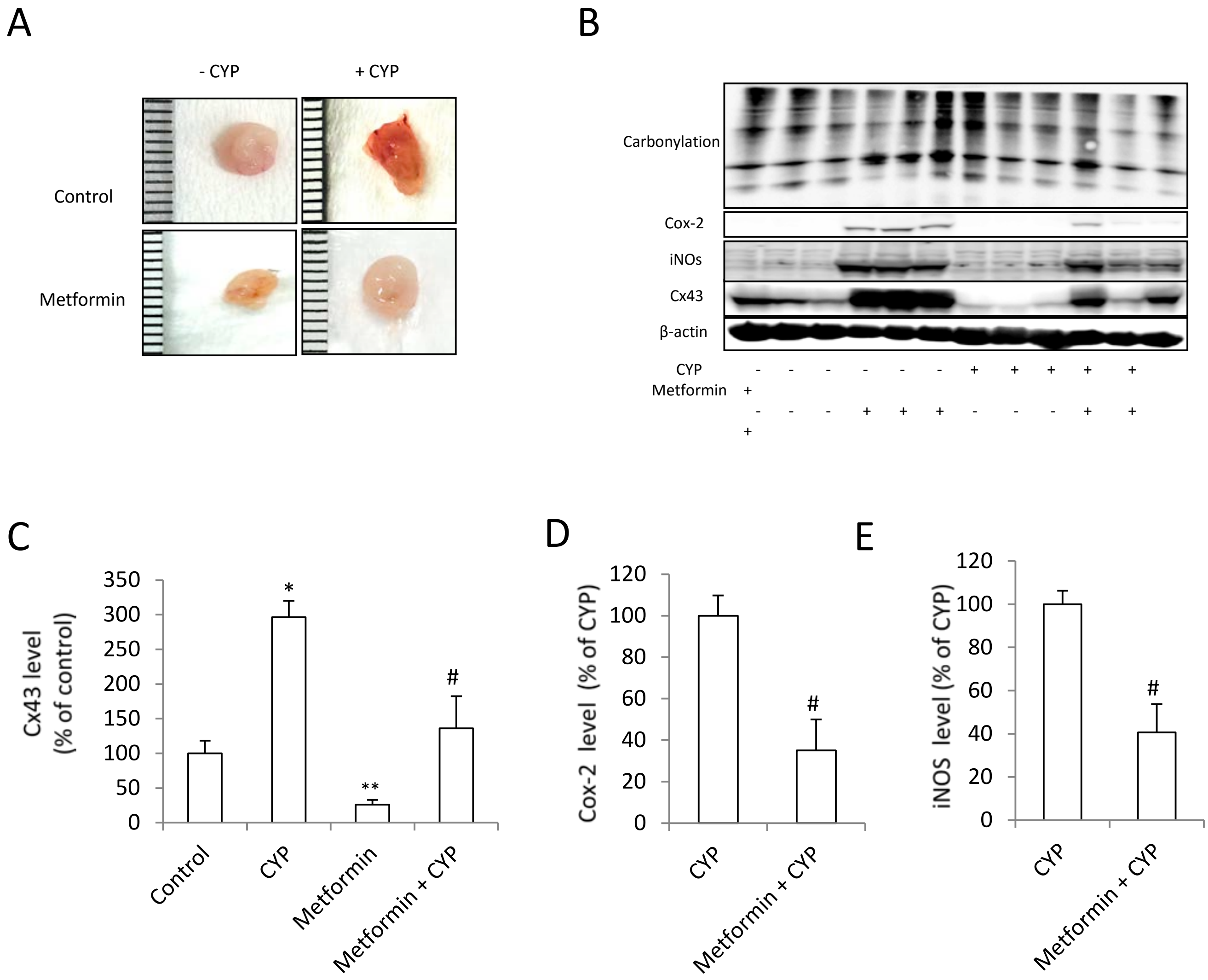

Supplement: Supplementary Information [file srep19708-s1.pdf]
